# Shade effects on growth, photosynthesis and chlorophyll fluorescence parameters of three *Paeonia* species

**DOI:** 10.7717/peerj.9316

**Published:** 2020-06-09

**Authors:** Yingling Wan, Yixuan Zhang, Min Zhang, Aiying Hong, HuiYan Yang, Yan Liu

**Affiliations:** 1 College of Landscape Architecture, Beijing Forestry University, Beijing, Beijing, P. R. China; 2 Management Office, Caozhou Peony Garden, Heze, Shandong province, P. R. China; 3 Beijing Laboratory of Urban and Rural Ecological Environment, Beijing, Beijing, P. R. China; 4 National Engineering Research Center for Floriculture, Beijing, Beijing, P. R. China

**Keywords:** *Paeonia anomala*, *Paeonia intermedia*, *Paeonia veitchii*, Low light intensity, Chlorophyll florescence parameters

## Abstract

Insufficient light intensity inhibits the growth of cultivated herbaceous peony and decreases its economic value. Owing to the increased demand for shade-tolerant herbaceous peony, the selection of appropriate parents for hybridization is essential. *Paeonia anomala*, *Paeonia intermedia* and *Paeonia veitchii* can grow under shade conditions in their natural habitats; however, their photosynthetic capacities under shade have not been studied. In this study, we simulated low light intensity (30% sunlight) and evaluated the morphological, photosynthetic and chlorophyll fluorescence parameters of these three species. Moreover, the shade tolerance of these species as well as two common cultivars (*Paeonia lactiflora* ‘Da Fugui’, which is suitable for solar greenhouse cultivation, and* P. lactiflora* ‘Qiao Ling’, which is not suitable for solar greenhouse cultivation) was evaluated. The results showed that under shade, the leaf area of *P. anomala* and *P. intermedia* increased, the single flowering period of *P. intermedia* and *P. veitchii* was prolonged, and the flower color of *P. veitchii* faded. With respect to *P. anomala*, *P. intermedia* and *P. veitchii*, shade eliminated the photosynthetic ‘lunch break’ phenomenon and decreased photoinhibition at midday. Furthermore, the maximum photochemical efficiency (Fv/Fm) and maximum primary photochemical yield (Fv/Fo) of photosystem II (PSII) in the three species improved significantly, and their changes in light dissipation were different. The shade tolerance of the tested accessions was in the order *P. veitchii* > *P. intermedia* > *P. anomala* > ‘Da Fugui’ > ‘Qiao Ling’, showing that the three wild species were better adapted to low light intensity than the cultivars. Thus, *P. anomala*, *P. intermedia* and *P. veitchii* could potentially be used in the development of shade-tolerant herbaceous peony cultivars.

## Introduction

Ornamental crops have high economic value because of the global trade of cut and potted flowers (Chandler & Sanchez, 2012; Prakash, 2007). Greenhouses have been widely used to produce specific ornamental products at desirable times. However, compared with those in the field, light changes in the greenhouse affect flowering time, stem length, and the number of branches and nodes of ornamental crop species (Bergstrand, 2017; Runkle & Heins, 2005; Stamps, 2009). In particular, the decrease in light intensity in the greenhouse is one of the most important factors affecting growth speed and biomass (Fini et al., 2010; Metsoviti et al., 2020). Thus, judging the shade adaptation or tolerance of plants in the natural environment can help future light adjustment strategies in greenhouses.

The economic value of the herbaceous peony (*Paeonia lactiflora*) is increasing in the worldwide market of ornamental plants (Kamenetsky & Shlomi, 2010). Growing in the field is the most common way for the cultivation of the herbaceous peony, but the short flowering season inhibits market availability, which cannot be offset by additional supply from the Southern Hemisphere (Kamenetsky & Dole, 2012). Accurate flowering regulation can be achieved via greenhouses; however, it has been suggested that the growth, flowering and stem straightness of the herbaceous peony are inhibited by decreased light intensity and photoperiod duration in the winter in solar greenhouses, which are widely used in China (Han et al., 2014; Zhao et al., 2015b). Preliminary studies have shown that only four of the main field-cultivated herbaceous peonies in China can survive in solar greenhouses (Wu et al., 2014). Thus, the development of shade-tolerant cultivars is urgently needed.

Breeding of shade-tolerant herbaceous peony is slow due to the narrow genetic background and same parental species (i.e., *P. lactiflora*) of the main cultivars (Kamenetsky & Dole, 2012). Interspecific hybridization is an effective way to transfer target traits to ornamental crop plants (Mii, 2009). One or several desirable traits, including flower type, color, flowering time and resistance to biotic and abiotic stresses, have been introduced to ornamental plants in the *Allium*, *Chrysanthemum*, and *Dianthus* genera (Cheng et al., 2011; Gatt et al., 1998; Nomura et al., 2002). Regarding the genus *Paeonia*, Itoh hybrids are the result of a successful interspecific hybridization of *Paeonia* species and present an improved flowering period and disease resistance (Page, 2005). However, to the best of our knowledge, no shade-tolerant herbaceous peony accessions have been developed. Given that the natural hybrid offspring between *Iris fulva* and *Iris hexagona* is shade tolerant (Bennett & Grace, 1990) and interspecific hybridization application creates many ideal ornamental cultivars, we may succeed to develop shade-tolerant herbaceous peony cultivars by interspecific hybridization techniques. As such, the first step is to identify the most suitable parent species.

*Paeonia anomala*, *Paeonia intermedia* and *Paeonia veitchii* are three species of sect. *Paeonia* for whom the edges of forests or sparse woods are their common natural habitats (Hong & Pan, 2004). Previous studies have mainly focused on the medicinal value of the extracts of these species or investigated these species from a phylogenetic perspective (Deyuan, Kaiyu & Turland, 2001; Kim et al., 2014; Pan, Zhang & Sang, 2007), and little attention has been paid to the photosynthetic characteristics of these three species in their original habitats (Jian et al., 2010). We found that some populations of *Paeonia anomala* can live under canopy shade, where light intensity at midday was only 156–237 µmol m^−2^ s^−1^ or 698–865 µmol m^−2^ s^−1^ at different locations (personal observation). A previous study showed that light intensity of a solar greenhouse was 30–1,000 µmol m^−2^ s^−1^ from 8:00–17:00 h (Han et al., 2014), while it reached 1,000 µmol m^−2^ s^−1^ and higher values in the field (Yue & Shi, 2010). It seems that these wild herbaceous peonies have the potential to adapt to the relatively low solar radiation of the greenhouse. Notably, plant canopies also decrease the radiation intensity of each waveband to different degrees, and this spectral composition change (e.g., R:FR ratio) affects morphological characteristics (Wherley, Gardner & Metzger, 2005; Zhang et al., 2016a; Zhang et al., 2016b). It is difficult to determine whether these three species can survive and maintain their shade tolerance under low light intensity when the ratio of red light to blue light is not altered (i.e., the light conditions of a solar greenhouse).

Under light stress, several morphological and physiological characteristics of plants change. The shade tolerance index is used to evaluate these characteristics for forest understory species (Humbert et al., 2007); however, the evaluation of shade tolerance can vary with plant type. For woody plant species, equations and traits for shade tolerance have been established, for example, indexes for wood density, sapwood area per leaf area and other traits that crop species do not exhibit (Falster, Duursma & FitzJohn, 2018). For crop species (e.g., soybean and potato), the membership function method has been used to evaluate shade tolerance, with indexes based on some photosynthetic and chlorophyll fluorescence parameters (Li et al., 2014; Liu et al., 2019), which are more suitable for evaluating the shade tolerance herein.

Failure to adapt to greenhouse light environments in most commonly field-grown peonies makes necessary the introduction of more shade tolerant genotypes, and *P. anomala*, *P. intermedia* and *P. veitchii* may act as potential parents. Our objective was to evaluate the shade tolerance of *P. anomala*, *P. intermedia* and *P. veitchii* under simulated solar greenhouse light conditions (low light intensity). We measured growth and flowering traits of these species under shade and compared their photosynthetic and chlorophyll fluorescence parameters with those of commonly cultivated herbaceous peony cultivars. We also utilized a membership function to classify the shade tolerance of these accessions. We hypothesized: (1) that *P. anomala*, *P. intermedia* and *P. veitchii* could survive under artificial low light intensity; (2) that their photosynthetic characteristics under shade would be similar to or even better than those under full sunlight; and (3) that their shade tolerance would be better than that of common cultivars. This study may provide a foundation for the selection of herbaceous peony parent cultivars, which would be helpful for cultivating hybrid progenies with improved shade tolerance.

## Materials & Methods

### Plant material and growth conditions

Five accessions were arranged in a completely randomized design, and the interval of each accession made sure the leaves of different plants were not covered. First, the three *Paeonia* species were introduced as plants with as much of the root system and underground buds as possible to the National Engineering Research Center for Floriculture, Changping district, Beijing, in August 2016. We used *P. anomala* plants (*n* = 17) from the Altay city population, *P. intermedia* plants (*n* = 24) from the Yumin population, Xinjiang Province, and *P. veitchii* plants (*n* = 22) from the Lanzhou population, Gansu Province. Deep, fertile and well-drained soil was selected for the field plantings. Before the seedlings were transplanted, the soil was tilled, stones and weeds were removed, and decomposed organic fertilizer (0.25 kg/m^2^ cake fertilizer) was applied. Seedlings were set apart 60 cm ×60 cm from the neighbor ones and were watered in accordance with the local weather conditions. Fertilizer was applied three times a year, that is, 1. 5 × 10^−2^ kg/m^2^ fertilizer NPK 30-10-10 in early spring after the soil thawed, 7. 5 ×10^−3^ kg/m^2^ fertilizer NPK 20-20-20 two weeks after flowering, and 7.5 × 10^−3^ kg/m^2^ fertilizer NPK 15-10-30 before the soil froze over after autumn. Weeding was performed throughout the growing season. After two years of cultivation in Beijing, more than 80% of these seedlings survived. In addition, *P. lactiflora* ‘Da Fugui’ (which is suitable for solar greenhouse cultivation; (Han et al., 2014)) and ‘Qiao Ling’ (which is not suitable for solar greenhouse cultivation), two common cultivars grown in China, were planted and managed as the wild *Paeonia* species.

**Figure 1:**
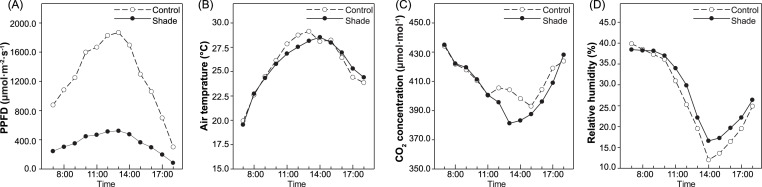
Environment factors in control group and under shade. Light intensity (A), air temperature (B), CO_2_ concentration (C) and relative humidity (D). All of these indicators were measured every one hour from 7:00–18:00h.

A single-factor experiment with each species was carried out in March 2018. Herbaceous *Paeonia* species needs to renew buds underground to germinate and develop crowns and flowers every year. Before germination in 2018, a black nylon net was placed above the planting location of the three species and two cultivars as shade treatment; under this net, the natural light experienced by the plants was approximately 30% of the sunlight intensity. Full sun exposure was used as a control treatment. Plants of each treatment received the same fertilizer and amount of watering. The daylength during the experiment was 12.21–14.86 h, and the average was 13.69 ± 0.80 h. The actual light intensity, air temperature, CO_2_ concentration and relative humidity above and below the shade net were recorded by a LI-6400 Portable Photosynthesis Measurement System (LI-COR, USA) with the measurement of the net photosynthesis rate (Pn). Concurrently, from 8:00 to 16:00 h, the light intensity under full sun exposure was greater than 1000 µmol m^−2^ s^−1^, while it was between 297.23–523.23 µmol m^−2^ s^−1^ at the same time under shade. The CO_2_ concentration was between 392.64–423.21 µmol mol^−1^ under full sun exposure and 385.52–426.53 µmol mol^−1^ under shade, respectively. Besides, the CO_2_ concentration under the shade net was significantly lower than that above the net from 12:00–14:00 h, and during that time, the temperature under the shade net was lower than outside it by approximately 2.22–2.86 ° C (Fig. 1).

### Morphological and floral measurements

Morphological traits were measured at flowering (i.e., 30–37 days after shading). Crown width, branch length, stem diameter, width and length of the third or fourth leaf from the top, and flower diameter were measured by a flexible ruler or a Vernier caliper, and we performed every measurement three times in three different individuals of each accession. In addition, the leaves were fully spread out on graph paper, and images were taken. The leaves were then outlined, and the leaf areas were calculated by Autodesk Computer Aided Design (AutoCAD, Autodesk, USA). Floral parameters, including flowering rate, flower number per pot and single flowering period duration, were recorded. Flower color was measured by a portable multifunction colorimeter (3nh, China). A D65 standard light source with an eight mm window diameter was selected as the measuring light source, and the outer surface of the petal was measured. The lightness (*L*^∗^), red/green coordinate (*a*^∗^) and yellow/blue coordinate (*b*^∗^) color values defined by the International Commission on Illumination (CIE) were recorded, and the measurements were repeated three times on different flowers.

### Photosynthetic measurements

Photosynthetic parameters were measured 20 days after the flowering of each accession, which was variable. The short time interval from germination to flowering and the energy store for vegetative propagation of the following year were considered in the selection of measurement time. Three plants were randomly selected per accession under conditions of full sun exposure and under shade to measure the photosynthetic parameters (using a LI-6400 Portable Photosynthesis Measurement System, LI-COR, USA), and three leaves (the third or fourth leaf from the top of plants in different stems) from each plant were measured. To obtain diurnal variation in photosynthesis, the Pn was measured every hour from 7:00 h to 18:00 h using a transparent leaf chamber, with three to six measurements per accession. During this process, stomatal conductance (Gs), intercellular CO_2_ concentration (Ci) and transpiration rate (Tr) were recorded simultaneously. To construct light response curves, the Pn under different levels of photosynthetic photon flux density (PPFD) (i.e., 2,000, 1,800, 1,600, 1,400, 1,200, 1,000, 800, 600, 400, 200, 150, 100, 50 and 0 µmol m^−2^ s^−1^) was measured from 8:30–11:30 h, with a CO_2_ concentration of 400 µmol mol^−1^. Three replicates were measured at each PPFD. Before the measurements, photosynthesis in the selected leaves was induced by 1,500 µmol m^−2^ s^−1^ PPFD for 20 min.

A nonlinear regression analysis was carried out according to the formula of the nonrectangular hyperbolic model, and a light response curve was generated. Linear regression of the Pn and PPFD in the range of 0–200 µmol m^−2^ s^−1^ was performed, and the apparent quantum yield (AQY), dark respiration rate (Rd), light-saturated photosynthesis rate (LSPn), light compensation point (LCP) and light saturation point (LSP) were calculated (Walker, 1989).

### Chlorophyll content and fluorescence measurements

During the flowering period, the third or fourth newest leaf under the flowers was randomly collected, and we used three leaves from three individuals per accession. After cleaning, 0.2 g of fresh leaves were cut into pieces, soaked in 25 ml of 95% ethanol and kept under dark conditions at room temperature. After 48 h, the absorbance of the solutions was measured at 665 nm and 649 nm by a Biomate 3S UV-visible spectrophotometer (Thermo Fisher Scientific, USA). The chlorophyll a and b contents were subsequently calculated by previously described equations (Alsaadawi, Al-Hadithy & Arif, 1986).

Chlorophyll fluorescence parameters were measured by a PAM-2500 portable amplitude modulation fluorometer (Walz, Germany) on the third or fourth leaf from each selected individual per accession. The minimal fluorescence with all photosystem II [PSII] reaction centers open (Fo), maximal fluorescence in the absence of NPQ in the dark-adapted state (Fm), minimal and maximal fluorescence in the presence of NPQ during illumination (Fo’ and Fm’) and steady-state fluorescence after onset of illumination (Fs) were recorded after 20 min of dark adaptation. To obtain Fo, a light pulse of 3 µmol m^−2^ s^−1^ was applied, and the modulation frequency was 20 kHz. To obtain the Fm, a saturating light pulse at an intensity of 8000 µmol m^−2^ s^−1^ was applied for 0.8 s. The light intensity during the measurement of Fo’ and Fm’ was determined according to the default program of the PAM-2500 portable amplitude modulation fluorometer.

Fv is calculated by the difference of Fm and Fo, and it reflects the reduction of electron acceptors of PSII(QA). The maximal PSII efficiency of dark-adapted leaves (Fv/Fm), maximum primary photochemical yield (Fv/Fo) of PSII, nonphotochemical fluorescence quenching (NPQ), quenching coefficient of photochemical quenching (q_p_) and relative PSII electron transport rate (ETR) were calculated according to various formulas (Demmig-Adams et al., 1996; Hu, Sun & Wang, 2007; Li et al., 2006). Similarly, the quantum yield of constitutive thermal energy dissipation (Φ_*D*_), quantum yield of PSII photochemistry (Φ_*PSII*_) and quantum yield of ΔpH- and xanthophyll-regulated thermal energy dissipation (Φ_*NPQ*_) were calculated according to the methods reported in previous studies (Hendrickson, Furbank & Chow, 2004; Zivcak et al., 2014).

### Evaluation of shade tolerance

The shade tolerance of plants is the result of many factors, and it cannot be judged from only a single index. The membership function method was used in conjunction with nine indexes of photosynthetic and chlorophyll fluorescence parameters to comprehensively evaluate the shade tolerance of the three wild *Paeonia* species and two cultivars. Fv/Fm is an acceptable parameter for evaluating whether a leaf is experiencing photoinhibition and its degree (Baker, 2008; Peng et al., 2017). Thus, we considered Fv/Fm a basic indicator for shade tolerance and calculated its correlation with five of the measured photosynthetic parameters (i.e*.*, the AQY, LCP, LSP, Rd and change rate of the LSPn under shade) and three chlorophyll fluorescence parameters (i.e., Fv/Fo, ETR and Φ_*PSII*_ change under shade) (Table S1).

The membership function method was used to evaluate the shade tolerance of plants according to methods of previous studies (Liu et al., 2019; Wang et al., 2014). Formula (1) was used if the index was positively related to Fv/Fm, and formula (2) was used if the index was negatively related to Fv/Fm.

(1) \begin{eqnarray*}{\mathrm{Z}}_{\mathrm{ij}}& =({\mathrm{X}}_{\mathrm{ij}}-{\mathrm{X}}_{\mathrm{i} \min \nolimits })/({\mathrm{X}}_{\mathrm{i} \max \nolimits }-{\mathrm{X}}_{\mathrm{i} \min \nolimits })\end{eqnarray*}

(2) \begin{eqnarray*}{\mathrm{Z}}_{\mathrm{ij}}& =({\mathrm{X}}_{\mathrm{i} \max \nolimits }-{\mathrm{X}}_{\mathrm{ij}})/({\mathrm{X}}_{\mathrm{i} \max \nolimits }-{\mathrm{X}}_{\mathrm{i} \min \nolimits })\end{eqnarray*}

Z_ij_ is the shade tolerance value of the i index for the j plant accession according to the membership function, and X_ij_ is the measured value of the i index for the j plant accession. X_i min_ and X_i max_ are the minimum and maximum values of each index, respectively. The membership function values of each index were averaged per accession. The higher the average value, the greater the shade tolerance of the plant.

### Statistical analysis

We compared every parameter under shade and sun exposure via the least significant difference method (LSD) after one-way ANOVA was performed (SPSS 18.0). Microsoft Excel 2016 and R 3.5.1 (R Core Team, 2019) were used to plot the results.

## Results

### Morphological and floral characteristics

**Figure 2:**
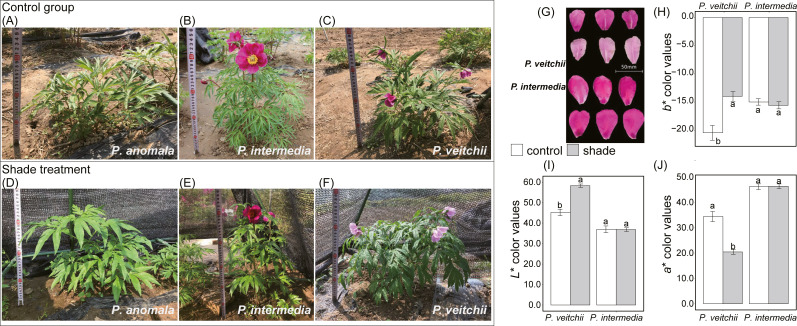
Morphological photos and flower color of three species of sect. *Paeonia*. (A–F) Photos of *P. anomala*, *P. intermedia* and *P. veitchii* under control and shade, respectively. (G) Flower colors of *P. intermedia* and *P. veitchii*. Three color indicators, (H) *b* ^∗^**, (I) *L* ^∗^** and (J) *a* ^∗^** color values, were measured by portable multi-function colorimeter (3nh, China).

The single flowering period of *P. intermedia* and *P. veitchii* was prolonged by shade, while their flowering rate and flower diameter were not affected (Table 1). *P. anomala* could not flower under any light condition in Beijing (Fig. 2). Moreover, the flower color of *P. veitchii* faded under shade, and it presented significantly higher *L*^∗^ and *b*^∗^ color values and lower *a*^∗^ values, showing an increase in lightness and a decrease in red and blue (Fig. 2). *P. anomala* and *P. intermedia* had larger leaf areas under shade than under full sun. No differences were observed in crown width, branch length or stem diameter for any of the three species under any light condition (Table 1).

**Table 1:** Morphological and floral characteristics of three species under sun exposure and shade treatments.

**Characteristics**	***P. anomala***		***P. intermedia***		***P. veitchii***	
	**Control**	**Shade**	**Control**	**Shade**	**Control**	**Shade**
Crown width (cm)	44.96 ± 5.67a	46.07 ± 6.53a	39.35 ± 9.17a	40.76 ± 7.91a	43.32 ± 8.75a	41.75 ± 7.16a
Branch length (cm)	24.08 ± 5.45a	25.26 ± 5.24a	48.51 ± 7.21a	47.93 ± 12.78a	30.26 ± 3.56a	29.72 ± 4.54a
Stem diameter (mm)	7.33 ± 1.30a	6.84 ± 1.51a	6.44 ± 0.86a	6.92 ± 1.47a	5.97 ± 0.89a	5.52 ± 0.87a
Leaf areas (cm^2^)	16.38 ± 3.84b	28.17 ± 1.54a	9.76 ± 1.22b	13.67 ± 0.53a	17.46 ± 0.73a	18.88 ± 0.53a
Flowering rate (%)	/	/	72.62 ± 18.05a	70.00 ± 24.01a	46.67 ± 5.09a	52.22 ± 6.51a
Flower amount per plant	/	/	3.00 ± 1.22a	2.60 ± 1.14a	2.04 ± 0.76a	1.76 ± 0.56a
Flower diameter(cm)	/	/	9.61 ± 1.26a	10.06 ± 1.53a	4.85 ± 0.82a	5.26 ± 0.54a
Single flowering period (d)	/	/	6.88 ± 0.75b	8.16 ± 0.75a	5.66 ± 0.89b	7.00 ± 1.75a

**Notes.**

Different lower-case letters showed significant difference (*p* < 0.05), while the same letters showed no significant difference. *P. anomala* did not blossom within two years of introduction in Beijing, and no blossom indicator was observed.

### Photosynthetic characteristics

**Figure 3:**
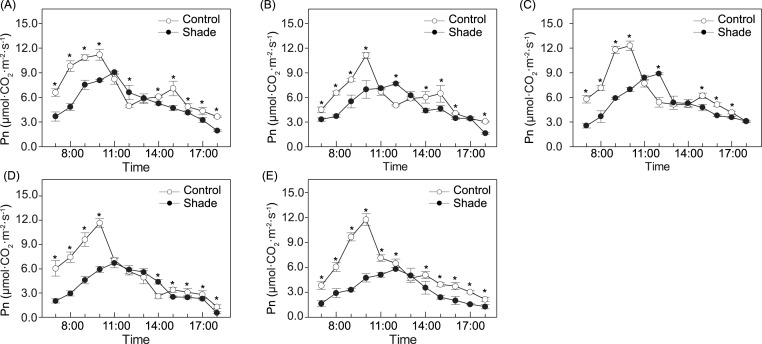
Photosynthesis diurnal variation in *P. anomala* (A), *P. intermedia* (B), *P. veitchii* (C), ‘Da Fugui’ (D) and** ‘Qiao Ling’ (E). The asterisks in the figures indicate there are significant differences between two light conditions (*p* < 0.05).

The photosynthesis diurnal variation of the three species was bimodally distributed under sun exposure, peaking at approximately 10:00 h and 15:00 h (Fig. 3). Under shade, single-peak photosynthesis curves were detected for the three species, and at those moments, Pn was significantly higher under shade than under sun. For the two cultivars, both the sun and shade groups presented single-peak curves, while peaking at 10:00 h under sun and at 11:00h or 12:00h, under shade, respectively. No significant differences were detected in ‘Da Fugui’ at midday between the two light conditions, and the Pn of ‘Qiao Ling’ under shade at midday was significantly lower than that under full sun. For all five accessions, the Pn in the morning (7:00–10:00 h) and afternoon (14:00–18:00 h) in the sun was often higher than that under shade.

**Figure 4:**
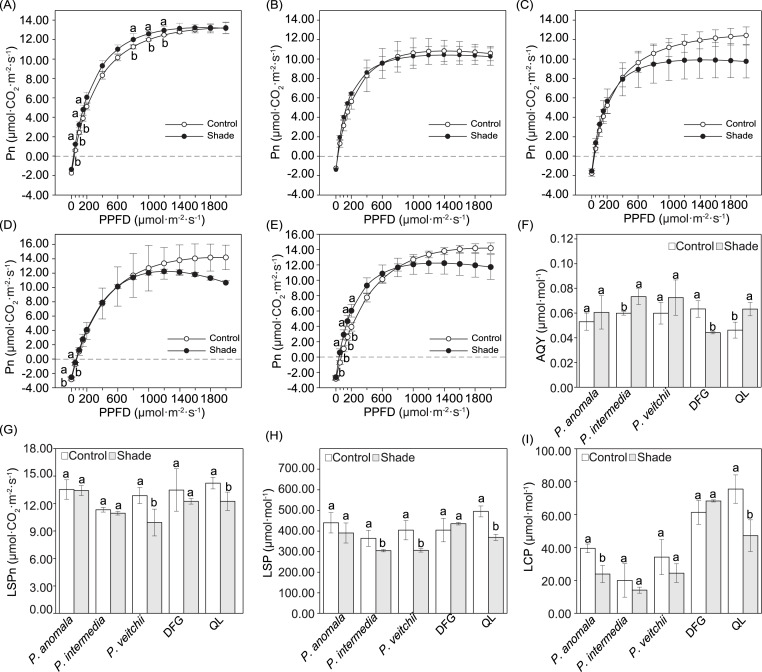
Light response curves and and related parameters calculated from the curves. Light response curves of *P. anomala* (A), *P. intermedia* (B), *P. veitchii* (C), ‘Da Fugui’ (D) and ‘Qiao Ling’ (E) and related parameters calculated from the curves. The (F) apparent quantum yield (AQY), (G) light-saturated photosynthetic rate (LSPn), (H) light-saturation point (LSP) and (I) light-compensation point (LCP) are shown. The different lowercase letters indicate significant differences (*p* < 0.05), while the same letters indicate no significant differences. In (A–E), to make the figures clearer, objects lacking significant differences were not marked with a lowercase letter a. In (F–I), DFG refers to ‘Da Fugui’, and QL refers to ‘Qiao Ling’.

The Pn increased linearly within the PPFD range of 0–200 µmol m^−2^ s^−1^, continuously increased at a lower rate in the PPFD range of 200–1,000 µmol m^−2^ s^−1^, and then remained unchanged or only slightly changed under higher PPFD (Figs. 4A–4E). Significant differences in the Pn between the two light conditions occurred under only a few light intensities. When the PPFD was 50–150 and 800–1.000 µmol m^−2^ s^−1^, the Pn of *P. anomala* under shade was significantly higher than that under sun exposure (Fig. 4A). For ‘Da Fugui’, differences between the sun and shade groups occurred only at 0 and 50 µmol m^−2^ s^−1^ (Fig. 4D), and when the PPFD was 20–200 µmol m^−2^ s^−1^, the Pn of ‘Qiao Ling’ was significantly higher than that under shade (Fig. 4E); in both cases, Pn increased.

The AQY significantly increased in *P. intermedia* and ‘Qiao Ling’ but decreased in ‘Da Fugui’ under shade (Fig. 4F). The LSPn, LSP and LCP decreased to different extents, with the exception of those of ‘Da Fugui’, which remained unchanged (Figs. 4G–4I). Among them, with respect to *P. anomala*, shade significantly decreased only the LCP (Fig. 4I); for *P. intermedia*, the LSP was significantly decreased in response to shade (Fig. 4H); and for *P. veitchii*, both the LSPn and LSP decreased significantly in response to shade (Figs. 4G–4H). All three parameters decreased in ‘Qiao Ling’ under shade (Figs. 4G–4I).

**Figure 5:**
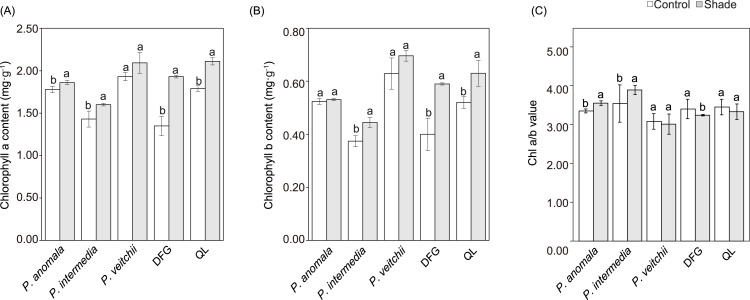
Chlorophyll a content (A), chlorophyll b content (B) and the ratio of chlorophyll a to b (Chl a/b) (C) of the three species. DFG refers to ‘Da Fugui’, and QL refers to ‘Qiao Ling’. The different lowercase letters indicate significant differences (*p* < 0.05), while the same letters indicate no significant differences.

The chlorophyll content tended to increase in response to shade, while the changes in chlorophyll a and b in *P. veitchii* and chlorophyll b in *P. anomala* were not significant. The Chl a/b significantly increased in response to shade in *P. anomala* and *P. intermedia*, whereas it significantly decreased in ‘Da Fugui’ (Fig. 5).

### Chlorophyll fluorescence characteristics

**Figure 6:**
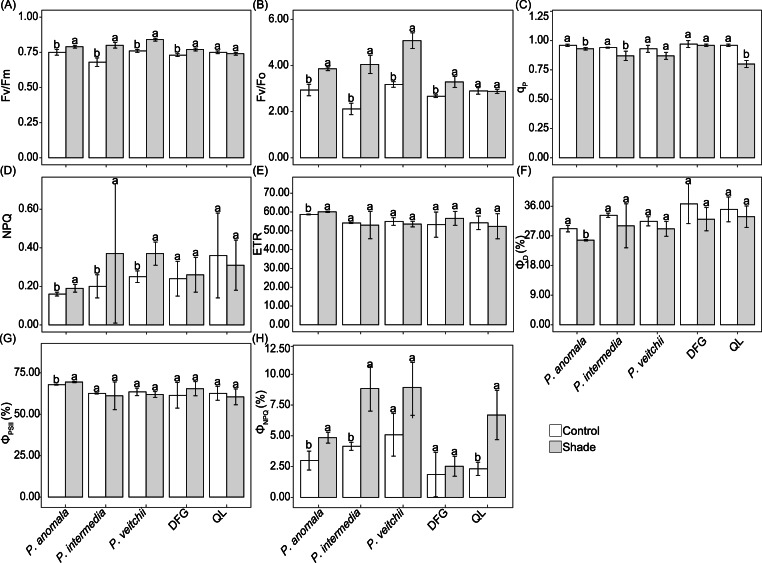
Five chlorophyll fluorescence parameters of five *Paeonia* accessions under two light conditions. (A) maximal PSII efficiency of dark-adapted leaves (Fv/Fm), (B) maximum primary photochemical yield of PSII (Fv/Fo), quenching coefficient of photochemical quenching (*q*_*p*_), nonphotochemical fluorescence quenching (NPQ) and relative PSII electron transport rate (ETR). The Fo values of *P. anomala*, *P. intermedia*, *P. veitchii*, ‘Da Fugui’ and ‘Qiao Ling’ in the control group were 0.28 ± 0.01, 0.32 ± 0.03, 0.28 ± 0.03, 0.13 ± 0.003 and 0.17 ± 0.004, respectively, and those in the shade group were 0.29 ± 0.01, 0.32 ± 0.02, 0.28 ± 0.02, 0.14 ± 0.01 and 0.17 ± 0.02, respectively. The distribution of light energy absorbed by the five accessions is shown in (F–H). (F): Quantum yield of constitutive thermal energy dissipation (Φ_*D*_), (G): quantum yield of PSII photochemistry (Φ_*PSII*_) and (H): quantum yield of ΔpH- and xanthophyll-regulated thermal energy dissipation (Φ_*NPQ*_). The different lowercase letters indicate significant differences (*p* < 0.05), while the same letters indicate no significant differences.

The Fv/Fm and Fv/Fo of *P. anomala*, *P. intermedia*, *P. veitchii* and ‘Da Fugui’ increased significantly in response to shade (Figs. 6A–6B). The NPQ of *P. anomala*, *P. intermedia* and *P. veitchii* increased, and the q_p_ of the last two accessions decreased significantly (Fig. 6C). With respect to ‘Qiao Ling’, only q_p_ decreased significantly in response to shade (Fig. 6C), and the Fv/Fm, Fv/Fo and NPQ remained unchanged (Figs. 6A–6B, 6D). Moreover, only the ETR of *P. anomala* increased with shade treatment (Fig. 6E).

Regarding the distribution of the absorbed light energy, both Φ_*D*_ and Φ_*PSII*_ of *P. anomala* decreased significantly in response to shade, and no significant differences were observed in these two parameters for the other four accessions (Figs. 6F–6G). The Φ_*NPQ*_ tended to increase under shade in all the samples, although the differences were significant only for *P. anomala*, *P. intermedia* and ‘Qiao Ling’ (Fig. 6H).

### Evaluation of shade tolerance of five accessions

The average scores of the three wild species were similar and significantly higher than those of ‘Da Fugui’ and ‘Qiao Ling’, indicating that the shade tolerance of *P. anomala*, *P. intermedia* and *P. veitchii* was greater than that of the common cultivars grown in China. In addition, the average score of ‘Da Fugui’ was slightly higher than that of ‘Qiao Ling’, but this difference was not significant (Table 2).

**Table 2:** Subordinate function values of the shade tolerance evaluation index based on photosynthesis parameters and chlorophyll fluorescence parameters. DFG refers to ‘Da Fugui’, and QL refers to ‘Qiao Ling’. ΦPSII and LSPn change refer to the change rate of the ΦPSII and LSPn under sunlight compared with shade, respectively.

**Accession**	**AQY**	**Fv/Fm**	**Fv/Fo**	**ΦPSII change**	**LSPn change**	**ETR**	**LCP**	**LSP**	**Rd**	**Average**
*P. veitchii*	0.65	0.94	0.91	0.30	0.27	0.57	0.78	0.94	0.77	0.68 ± 0.05 a
*P. intermedia*	0.67	0.55	0.50	0.30	0.85	0.53	0.97	0.94	0.84	0.68 ± 0.11 a
*P. anomala*	0.48	0.44	0.44	0.41	0.99	1.00	0.84	0.51	0.96	0.67 ± 0.05 a
DFG	0.02	0.32	0.20	0.67	0.88	0.88	0.02	0.06	0.30	0.37 ± 0.06 b
QL	0.44	0.07	0.04	0.28	0.54	0.49	0.38	0.50	0.19	0.33 ± 0.06 b

## Discussion

In this experiment, 30% sunlight had no significant effect on the flowering rate, crown width, branch length or stem diameter of *P. anomala*, *P. intermedia* and *P. veitchii*. A previous study showed that the height and stem diameter of *P. lactiflora* ‘Da Fugui’, which is a commonly cultivated herbaceous peony, decreased under 40% shade (Zhao, Hao & Tao, 2012). For this reason, it seems that *P. anomala*, *P. intermedia* and *P. veitchii* were more adaptable to low light intensity than ‘Da Fugui’. A decrease in size and branch number and an increase in stem length occurred in *Kalmia latifolia* (Brand, 1997), *Narcissus* and *Tulipa* (Cavins & Dole, 2002) in response to shade, but these phenomena were not observed in this study. Combined with the increase in leaf area, these results suggest that the wild *Paeonia* species used in this study exhibit different morphological responses to shade. *P. anomala* did not flower during the experimental period, which may be caused by other factors beyond light and requires further study. The single flowering period of both *P. intermedia* and *P. veitchii* was significantly prolonged under shade, and the latter also showed a faded color in its flowers. This is in accordance with recent studies that suggested that anthocyanin biosynthesis is affected by light quality (An et al., 2020), and appropriate blue and red ratios produced ideal plant colors (De Keyser et al., 2019). One possible explanation for the color change observed in our study would be that the wavelength range and light quality filtered by the canopy above *P. veitchii* in its native habitat is more suitable for the growth of this species.

Photosynthesis efficiency can be judged by changes in photosynthesis parameters and chlorophyll content (Hu, Sun & Wang, 2007). Under control conditions, the three species showed the so-called ‘lunch break’, which corresponds to the decrease in Pn at midday. Decreased air humidity and increased temperature are related to this phenomenon (Peng et al., 2015), which was consistent with our recorded environmental factors. In addition, the ‘lunch break’ phenomenon is also a characteristic of the shade-tolerant species *Hosta* (Zhang et al., 2004). During the stages of Pn decrease, the Gs and Tr of the five accessions decreased, and Ci increased (Table S2), so the main limitation of Pn for these herbaceous peonies was nonstomatal (Farquhar & Sharkey, 1982).

From the light response curve with a PPFD between 20 and 200 µmol m^−2^ s^−1^, the Pn of ‘Qiao Ling’ under shade was significantly higher than that under full sun exposure, which is consistent with its AQY change. The AQY reflects the light energy conversion efficiency of the photosynthesis apparatus and the photosynthesis capacity at low light intensity. A higher AQY indicates a stronger ability of plants to use low light (Richardson & Berlyn, 2002). Moreover, the reduction in the LCP and LSP under shade is the result of adaptations to environmental changes (Boardman, 1977). ‘Qiao Ling’ had enhanced AQY and reduced LCP and LSP under shade. However, the wild species had not consistent patterns (two of them did not show significantly higher AQY under shade and at least one of them did not change LCP or LSP). Moreover, the changes in chlorophyll content were consistent with the measured photosynthesis parameters. Previous studies have suggested that chlorophyll contents increase under shade, and Chl a/b decreases due to the higher increase in chlorophyll b than chlorophyll a with the goal of increasing the light absorption ability in the wavelength range between blue and red (Bertamini, Muthuchelian & Nedunchezhian, 2006; Boardman, 1977; Zhao et al., 2015a). Our results showed that the Chl a/b of the three species increased or remained unchanged, which is inconsistent with the trends exhibited by shade-tolerant forest plant species (Zivcak et al., 2014). A possible explanation for this is based on the constant proportion of red and blue light under shade in this experiment, which differed from the relatively low proportion of red light under the tree canopy (Zhang et al., 2016a; Zhang et al., 2016b).

Chlorophyll fluorescence parameters can reflect the degree of environmental impact on the plant photosynthesis apparatus (Rascher, Liebig & Lüttge, 2000). Fv/Fm is an indicator of damage in the photosynthetic apparatus or abiotic stress in leaves, and the standard value is c. 0.83 for non-stressed leaves (Baker, 2008). Our results showed that shade significantly improved Fv/Fm for *P. anomala*, *P. intermedia*, *P. veitchii* and ‘Da Fugui’, and their values were 0.84, 0.80, 0.79 and 0.77 under 30% sunlight, respectively. This result indicated that exposure to 100% sunlight caused photoinhibition in these three species and ‘Da Fugui’, and the imposition of 30% sunlight could possibly be insufficient for *P. intermedia*, *P. veitchii* and ‘Da Fugui’ to reach 0.83. In the shade-adapted species *Torreya grandis*, a similar phenomenon by which Fv/Fm increased under shade was observed (Lin et al., 2019), suggesting that *P. anomala*, *P. intermedia* and *P. veitchii* have some characteristics of shade-tolerant plants. Generally, light stress leads to an increase in NPQ and causes oxidative damage and the destruction of the PSII reaction center, associated with an increase in Fo (Baker, 2008). In our experiment, the Fo remained unchanged under shade, although the NPQ of *P. anomala*, *P. intermedia* and *P. veitchii* increased, indicating that shade did not damage their photosynthetic apparatus. Moreover, the q_p_ of *P. anomala*, *P. intermedia* and *P. veitchii* tended to decrease under shade. In addition, the ETR of *P. anomala* significantly increased under shade, indicating different response to shade for these three wild species.

Some light energy absorbed by plants is used for photosynthetic electron transport, and a large amount of energy is dissipated (Endo et al., 2014). We observed in *P. anomala* that shade increased the amount of energy used for photochemical reactions and decreased the amount that is thermally dissipated, as seen from the change in Φ_*PSII*_ and Φ_*D*_, suggesting an adaptation of *P. anomala* under shade. With respect to *P. anomala*, *P. intermedia* and ‘Qiao Ling’, the yield for dissipated energy from the nonphotochemical reactions in the reaction centers (Φ_*NPQ*_) increased under shade, showing that the plants can protect the PSII reaction centers by increasing nonphotochemical dissipation (Öquist et al., 1992). Moreover, it was suggested that plants can compensate for the decrease in Φ_*PSII*_ by increasing the ETR to ensure photosynthesis productivity (Hu, Sun & Wang, 2007). This was consistent with the trends of *P. anomala*, indicating that adaptive photochemical mechanisms in response to shade effects are highly developed in this species. However, the Φ_*PSII*_, Φ_*D*_ and Φ_*NPQ*_ of *P. veitchii* remained unchanged under both light conditions; thus, additional studies are needed to investigate the response to shade.

The comprehensive evaluation of shade tolerance by the membership function showed that the three wild species (i.e., *P. anomala*, *P. intermedia* and *P. veitchii*) had significantly higher scores, thus, they had better shade adaptation abilities to shade than the two commonly grown cultivars, which was consistent with the changes in both photosynthetic and chlorophyll parameters and was in agreement with our hypothesis. Several studies of shade tolerance in crops have applied principal component analysis (PCA) before using the membership function method (Liu et al., 2019; Wu et al., 2015), while other studies have suggested that if only several candidate evaluation indexes participate in the calculation of the membership function, methods without PCA perform better because the results with PCA preprocessing may be opposite to the actual performance of plants (Zhang et al., 2016a; Zhang et al., 2016b). The nine parameters we used were previously suggested to be relevant in the response to shade (Baker, 2008; Ntawuhiganayo et al., 2019; Pires et al., 2011), and the parameters that may be related to changes in light (e.g., NPQ) or were not consistent with previous studies (e.g., changes in chlorophyll content) were not selected. The results were satisfactory, showing the feasibility of the membership function method for evaluating plant shade tolerance.

## Conclusions

*P. anomala*, *P. intermedia* and *P. veitchii* grew well under 30% sunlight, with an overall increase in leaf area and length of the flowering period. However, *P. anomala* did not flower during the experimental period. Moreover, shade treatment (30% sunlight) can release photoinhibition caused by full sun exposure at midday. Comprehensive evaluation by the membership function showed that the shade tolerance of these three species was greater than that of cultivated herbaceous peonies and that these species. Thus, these species could potentially act as parents of hybrid herbaceous peonies.

##  Supplemental Information

10.7717/peerj.9316/supp-1 Table S1 Correlation matrix of selected photosynthesis index and chlorophyll fluorescence parameters

10.7717/peerj.9316/supp-2 Table S2 Stomatal conductance (Gs), intercellular CO_2_ concentration (Ci) and transpiration rate (Tr) of five accesions

10.7717/peerj.9316/supp-3 Supplemental Information 1 Raw data of enviroment indicators, petal color, photosynthetic and chlorophyll fluoresence parameters

## Additional Information and Declarations

### Data Availability

The following information was supplied regarding data availability: The raw measurements are available in the Supplemental Files.
